# Effects of synaptic connectivity inhomogeneities for propagation of activity in neural tissue

**DOI:** 10.1186/1471-2202-13-S1-P76

**Published:** 2012-07-16

**Authors:** Jie Zhang, Remus Osan

**Affiliations:** 1 Department of Mathematics and Statistics, Georgia State University, Atlanta, GA, 30303, USA; 2 Neuroscience Institute, Georgia State University, Georgia State University, Atlanta, GA, 30303, USA

## 

The study of traveling waves of activity in neural tissue can provide deep insights into the functions of the brain during sensory processing or during abnormal states such as epilepsy, migraines or hallucinations. Computational models of these systems usually describe the tissue as a vast interconnected network of neurons comprised of large number of units with similar properties, for example integrate and fire neurons. It is also widely assumed that while the strength of the connections between neurons changes as a function of distance, this interaction does not depend on other local parameters.

These assumptions allow for formulation of a set of integro-differential equations describing the propagation of the traveling wave fronts in a one-dimensional integrate-and-fire network of synaptically coupled neurons, allowing for investigation of the network dynamics during wave initiation and propagation. Equations for the transition between initiation and transition toward constant speed traveling waves have been derived for Gaussian connectivity [1] and finite support connectivity [2]. These results have been also confirmed through numerical simulations, leading to methods for optimizing and improving simulations of large-scale networks [3]. These results have been extended beyond the simpler case of one-spike activity propagation, deriving equations for constant speed waves with a finite and infinite number of spikes [4]. This framework has produced insight on the mechanisms of stable constant-speed traveling wave solutions, but the study of inhomogeneities in synaptic connections likely to exist in the brain tissue has received much less attention since not surprisingly, the presence of inhomogeneities vastly increases the complexity of the mathematical models. However, recent work [5,6] used homogenization theory to determine how inhomogeneities can induce propagation failure.

We extended our previous models that exhibit constant-speed traveling waves to investigate how the presence of these inhomogeneities affects the relationship between the speed of the activity propagation and its acceleration. We determine that the estimates from homogenization theory do not accurately capture the conditions for propagation failure. More precisely, just prior to stopping, the activity propagates at a higher average speed than predicted from the theoretical results of the homogenization theory. We derive more precise estimates for the conditions when propagation failure occurs. Furthermore, our study points to directions where researchers can obtain additional tools for analyzing experimental data in order to infer details of synaptic connectivity.
